# Supplementary material for: Complexome profiling of the Chlamydomonas psb28 mutant reveals TEF5 as an early PSII assembly factor
Source: Plant Cell. 2025 Mar 17;37(6):koaf055. doi: 10.1093/plcell/koaf055 (PMC12164590; doi:10.1093/plcell/koaf055)
Supplement: koaf055_Supplementary_Data [file koaf055_supplementary_data.zip › psb28_interactive.zip!/plots/intensity/qualityPlots/peptides/PRX4_Cre02.g080900.t1.2.html]

### Cre02.g080900.t1.2

Trivial name: PRX4  
SeqCoverage [median]: 17.30  
Mass: 24.37 kDa  
  
 

| Sequence | MissedCleavage | Mass [Da] | wtIntensity | muIntensity | Score |
| --- | --- | --- | --- | --- | --- |
| LLGLEIGAPEAGQGPK | 0 | 1548.85 | 556.16 | 0.00 | 100.93 |
| LRVESAPADLK | 1 | 1197.67 | 392.78 | 0.00 | 36.68 |
| YAAVVEDGVLLK | 0 | 1275.71 | 2119.44 | 7322.26 | 140.16 |
|  |  |  | 3068.4 | 7322.3 |  |
